# The Construction of Interactive Teaching Quality Monitoring System From the Perspective of Psychology

**DOI:** 10.3389/fpsyg.2022.849528

**Published:** 2022-02-22

**Authors:** Kewei Chen

**Affiliations:** Department of Educational Administration, Zhejiang Yuexiu University, Shaoxing, China

**Keywords:** interactive teaching, teaching monitoring system, B/S system, psychological perspective, teacher–student relationship

## Abstract

The article first proposes a reflection on the status quo of classroom teaching in public universities, selects a large number of educational scenes, and analyzes these selected educational concepts in detail from a theoretical perspective. Through the establishment of a teaching quality monitoring system based on careful observation and analysis, several major problems in public university classrooms have been discovered: poor classroom interaction mode, single classroom interaction mode, low classroom interaction efficiency, and inefficient classroom interaction feedback. Specific manifestations include the alienation of teacher–student relationship, insufficient student coverage, lack of interaction channels, lack of interaction context, only formalized teacher–student interaction, low impact, and simplification of teacher–student interaction. Summarizing the causes of classroom interaction problems, the article summarizes three factors: teachers, students, and the macro environment. In the experiment, 85% of the students thought that the teacher–student relationship was relatively ordinary. Among the 11 students who participated in the experiment, 60% of the students were very active in interactive classroom discussions. Therefore, based on the analysis of problems and factors, the article puts forward an optimization strategy to create a classroom interaction atmosphere, improve classroom interaction mode, improve classroom interaction efficiency, and strengthen classroom interaction feedback. In particular, it promotes the integration of teacher–student relationship, emphasizes the role of students, improves the level of classroom interaction, creates conditions for classroom interaction, improves teacher–student interaction control ability, enhances student interactive speech ability, strengthens error correction and feedback, and increases multiple feedback methods.

## Introduction

With the rapid development of online education such as mobile teaching, major changes have taken place in the educational concepts of colleges and universities. For students, in the “Internet + education” today, the demand for high-quality curriculum resources is increasing, and the task of sharing curriculum resources between universities vital. The emergence and formation of interactive teaching conforms to the progressive trend of the academic era and solves the problem of unbalanced resource allocation. The interactive teaching method has been researched and recognized by scholars. Interactive teaching should not be used for classroom teaching alone. For many years, educators have created interactive teaching models based on different platforms based on various platform researches. Teachers can understand students' learning status and learning progress through multiple platforms, and can communicate across regions. It is also possible to communicate and interact through online platforms to understand students' ideas, find their own shortcomings and correct them in time, and download teaching resources, and shared books to teachers through the network platform, so that students can learn on time. Classroom preview and interactive teaching methods enable teachers to teach better and students to learn more.

In terms of interactive teaching and quality monitoring system construction from the perspective of psychology, experts at home and abroad have also made many research results. Tho recognizes the role of signals in teaching quality evaluation and the advantages of set theory methods in educational research. The purpose is to use signal framework and fuzzy set qualitative comparative analysis (fsQCA) to configure the role of signal quality, including signal consistency, signal clarity, signal credibility, and teaching input in teacher teaching quality (Tho, 2017). Mbise's research shows that professional certification plays an important role in the teaching quality of teachers. Certification improves teaching quality, enhances self-confidence, keeps professional skills updated, improves the preparation of teaching plans and teaching materials, enhances teaching skills, and improves learning activity settings (Mbise, 2021). Wanpen and Somjinda requires the experimental group and the control group to complete their studies and test their perception ability. It is worth noting that theoretical teaching through interactive teaching methods can improve students' academic performance, perception ability, learning disaster response, and nursing action ability (Wanpen and Somjinda, 2017). Hundt et al. discussed a series of well-known parallel algorithms based on C++11 threads, OpenMP, MPI, and CUDA. These algorithms can use a unified source code to automatically evaluate interactive teaching methods and embed them in HPC or parallel computing lectures, that is, “automatic code Evaluation System” (SAUCE) (Hundt et al., 2017). Souzai et al. introduced the experience of university health psychology, emphasizing the practice, challenges, and relevance of this resource in cultivating future psychologists, and made contributions to the local population through health education (Souzai et al., 2019). Breda et al. has researched and proved that the teaching applicability standard proposed by the theoretical framework called the Ontology Notation Method of Mathematical Knowledge and Teaching (OSA) has become a powerful tool for teaching reflection and evaluation of the teaching process (Breda et al., 2017). However, although these studies are relatively comprehensive, there are some controversies in the methods used, resulting in related results that are not recognized by the public.

“Internet+” breaks the phenomenon of knowledge closure, and allows education to move from “closed school” to “renew and open.” It is very important to realize resource sharing. In the “Internet + education” today, teachers have transformed from teaching resources to teaching resource organizers, using multimedia to provide students with satisfactory learning resources and cultivate students' new thinking. In the era of “Internet + Education,” the demand for teachers' information literacy is increasing. Students can choose the courses they are most interested in through the platform and access the mobile platform anytime and anywhere. Teachers and students can communicate across spaces, emphasizing the diversity of teaching methods. In addition, there is a high demand for the quality and quantity of educational resources. The large-scale sharing of high-quality educational resources will benefit many scholars and eliminate the uneven distribution of resources. The significance of the construction of “Internet + education” to the development of education today is irreplaceable. On the one hand, colleges and universities can share teaching resources, solve the current situation of insufficient curriculum resources in colleges and universities, meet the needs of teaching, and avoid the waste of resources. Teachers can discuss together to provide more high-quality curriculum resources. On the other hand, students can choose courses to study on the platform according to their own actual situation and hobbies. Students can also conduct online interactive communication to expand their professional knowledge at one time and achieve personalized teaching.

## System Structure Model Analysis

College informatization is a major trend in the reform of teaching management in colleges and universities. Under the conditions of information and network, establishing a structure-based educational management information system that meets the requirements of the knowledge economy era, using information technology to transform traditional teaching and educational management, and realizing the leap-forward development of teaching and educational affairs is one of the important tasks of the current digital campus. Breaking the cumbersome and repetitive information transmission between various departments and departments through information construction, to enable information to be transmitted between the upper and lower levels of the school, between the school and the teacher, and between the teacher and the student in the fastest electronic way, and serve the school's teaching and scientific research. This will greatly improve the transparency of the school's various departments and departments, reduce office costs, save office hours, and improve work efficiency. This is very important for the school's democratic and scientific decision-making. With the development of education and technology, the reform of higher education is imperative, with the continuous deepening of the reform of the flexible academic system and the credit system. Many problems need to be solved, such as course selection, teacher–student communication, score query, etc., which cannot be solved by traditional manual methods. Therefore, many non-traditional teaching and management modes, such as online course selection, online examination, online inquiry of results, online teaching, online Q&A, online experiment, etc., must be fully implemented using network-based computer software systems. Information islands are formed between various systems, which increase the workload of users. Some schools have developed more complete systems. But the development cycle is very long, generally 1–2 years, and the system can only adapt to this school. Mature foreign university management software cannot adapt to the management situation of Chinese universities, and there are no mature products in China. We have the responsibility to develop an educational administration management system for universities and colleges that tracks the internationally leading level in terms of concepts and technologies.

### B/S+Soap Structure Model

**Figure 1:**
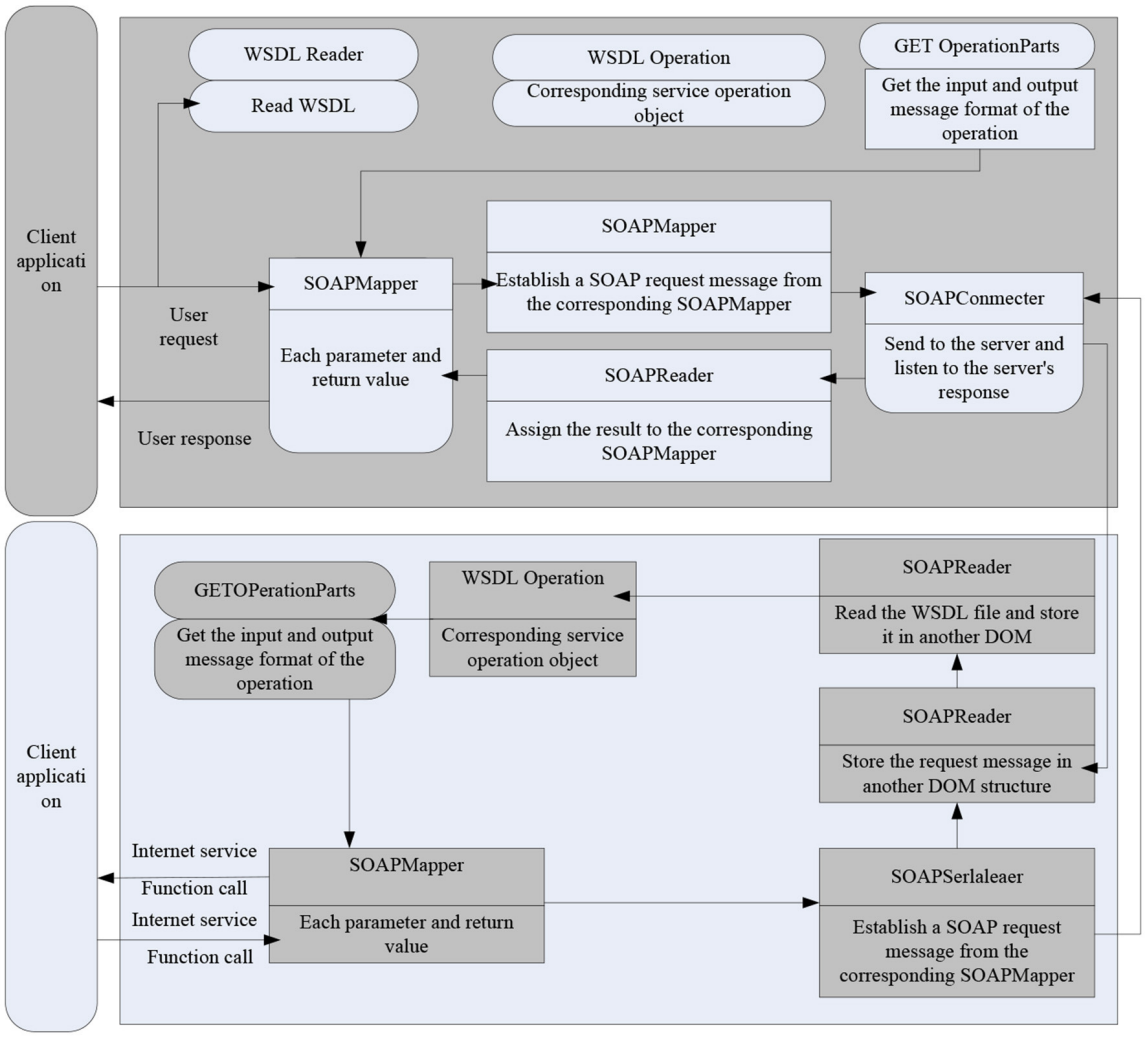
B/S+SOAP structure.

After several years of application, the structure of the B/S system has also exposed many shortcomings, the more prominent ones are the following:

(1) Because the browser is specially designed for WEB browsing, if it is used in a WEB application system, many functions are difficult to execute or understand (Fabisiak, 2018). For example, it may be more difficult to enter large amounts of data or respond to reports through a browser.
(2) The application structure is complex and difficult. Compared with a series of application tools built on more complex C/S, even if ActiveX, Java, and other technologies can be used to create more complex applications, but these technologies are complex and lack fully mature technologies to use.
(3) The low reliability of HTTP will cause application failures, especially for administrators, it is insecure and difficult to use browsers to maintain the system.
(4) The WEB server becomes the only client of the database, and all database connections can be accessed through this server. The WEB server must process client requests and database connections at the same time. The more visits, the greater the overload of the server.
(5) Business logic and data access programs are usually run by small programs embedded in JavaScript, VBScript, etc., scattered on different pages, difficult to share, difficult to upgrade, and maintain. At the same time, because the open source code is open, business rules are very important for applications. In order to overcome the above shortcomings, the SOAP mechanism is introduced in the B/S structure, which can distribute server-side functions to different machines, thereby achieving weight balance. In addition, the original server is now used as a SOAP client, further simplifying its development process. The SOAP client protects the complexity of task execution, and the use of SOAP to perform long-distance calls is to complete the communication between the client and the client on the same or different platforms. That is, the client sends a SOAP request, the client accepts the request, analyzes the information contained in it, calls the corresponding function, sends the return value to a SOAP, and finally the client interprets and responds to the message. Figure 1 shows the B/S +SOAP structure.

### The Overall Structure of the System

**Figure 2:**
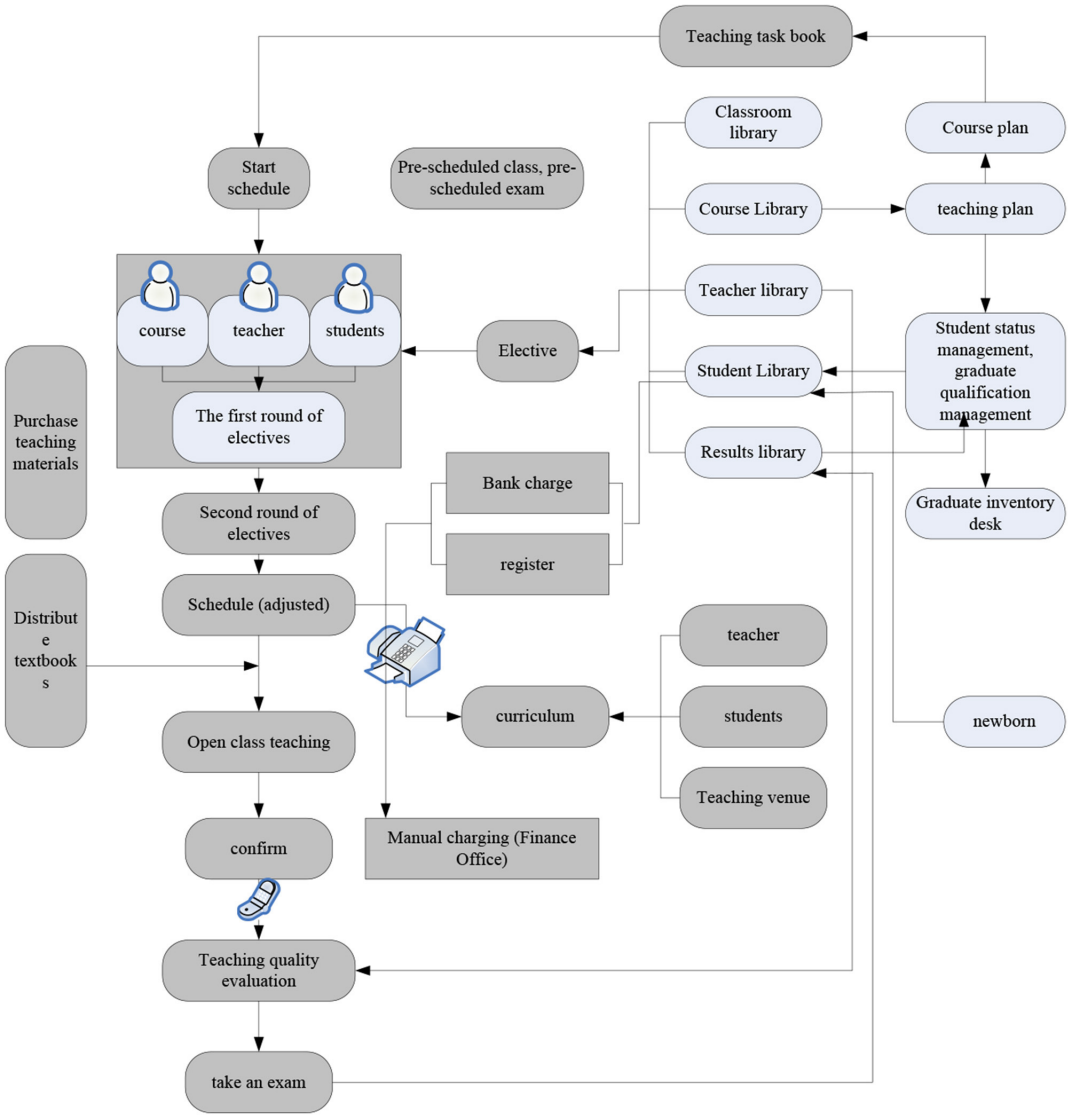
Academic Management System flow data and the module framework.

The basic management of the system takes the decision-making department of the Academic Affairs Office (the Academic Affairs Office, etc.) as the control center, centralizing and unifying all related data, and other departments (colleges, departments, teaching and research rooms, courseware rooms, etc.) are responsible for auxiliary work. After obtaining permission, they can access, edit, query, count, and print data. In this way, many activities of the Ministry of Education (Acquisition and update of student status information, performance management, teacher management, workload accounting, teacher evaluation, teaching plans, teaching materials design, student selection, degree acceptance and questioning, timetables, examination questionnaires, etc.) are divided into individual units, so that it can process data accurately and in a timely manner. The business process is shown in Figure 2. The data processing model of the system takes the teaching plan as the core, combines student status data and teacher data, automatically generates teaching basic data and teaching data design materials, presents them to students, creates schedule data, and processes exams data (Chen et al., 2018). Standardizing educational administrative business processing methods, improve processing efficiency, provide high-quality services for teaching management, provide a solid foundation for transformation, and provide services and comprehensive support for management decision-making.

**Figure 3:**
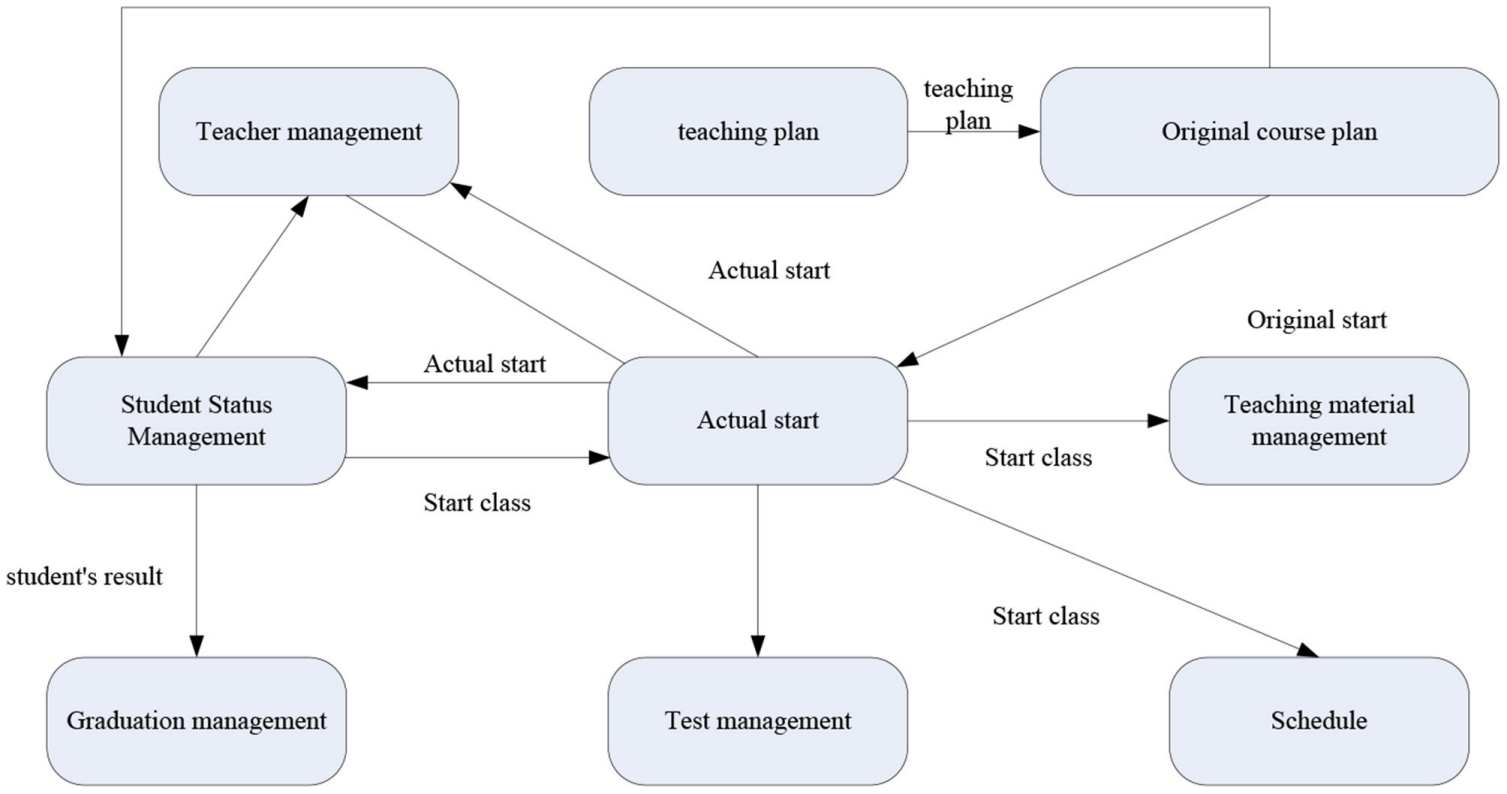
The data flow between the modules map.

The system is constructed based on a high starting point and high quality. It has the characteristics of friendly interface, easy to master, complete functions, functional safety and reliability, and multiple application functions, taking into account current adaptation and future development. The main functional modules include system maintenance, teacher management, teaching plan management, intelligent lesson scheduling, examination management, course selection management, performance management, teaching quality management, degree management, teaching material management, etc. Figure 3 shows the data flow diagram between the modules.

### Introduction to System Database

There are many current database products. Among them, the SQLServer2000 database system is a database management system with very good connectivity, which can be installed on WindowsNT/2000/Workstation/9X, but not on the network of other database systems. Therefore, whether it is a business-level application based on Windows NT/2000 Server or a small desktop application, the SQL Server 2000 database system provides users with comprehensive database support. The SQLServer2000 database system manages two types of databases: system database and user database, also known as SQLServer, dedicated to managing self and user databases, the database system generated by SQLServer includes Master, Model, Tempdb, Msdb, and Pubs and Northwind database samples are automatically generated for learning and use. The information of all students, teachers, and various resources in the system are stored in the database of the campus server to achieve system integrity and information sharing. Establishing a service database is the key to starting this system, and it is also the common advantage of this system and campus network. According to the guiding system thought, design principle, and demand analysis, the data related to the system is analyzed in detail, and the data table structure and definition are described according to the standardization requirements of the data. The following is a part of the field structure of some data of this system. Table 1 is the basic information of students, and Table 2 is the professional code table.

**Table 1:** Students basic information table.

**Field name**	**describe**	**Type of data**	**Is it empty**	**Remark**	**Numerical value**
XH	Student ID	Char (8)	Not null	Key	23
XSXM	Name	Varchar (20)	Not null		34
XSXB	Gender	Char (2)	Not null	Deafult (boy/girl)	23
CSRQ	Date of birth	Datetime	Not null		
ZZMM	Political status	Varchar (10)	Not null		
MZ	Nationality	Varchar (IO)	Not null		231
JG	Jihe	Varchar (20)	Not null		
BJBH	Class number	Char (6)	Not null		
RXRQ	Enrollment date	Datetime	Not null		23
SFZH	Identity number	Char (18)	Null		
WPDW	Appoint training unit	nVarchar (50)	Null		
XI	Tie	Varchar (20)	Null		34
LQH	Admission number	Char (8)	Not null		
ZYDM	Professional code	Char (6)	Not null		23
XXNX	Years of study	Int	Not null		23
XJZT	Student status	Char (2)	Not null	Deafult (yes/no)	67
DZZCH	Register Number	Char (8)	Not null		52

**Table 2:** Professional code table.

**Field name**	**Describe**	**Type of data**	**Is it empty**	**Remark**	**Number of people**
ZYDM	Professional code	Char (4)	Not null	Key	125
ZYMC	Professional title	Varchar (50)	Not null		254
XZ	School system	int	Not null		245
ZYPYMB	Professional training goals	Varchar (100)	Null		245
ZYKC	Professional Courses	Varchar (100)	Not null		254
TSMC	Featured name	Varchar (100)	Null		354
SSXDM	Department code	Char (2)	Not null	Foreign Key	87
ZYJC	Professional Abbreviation	Varchar (20)	Null		42
ZYYWMC	Professional English name	Varchar (50)	Null		354

### System Logical Structure Design

**Figure 4:**
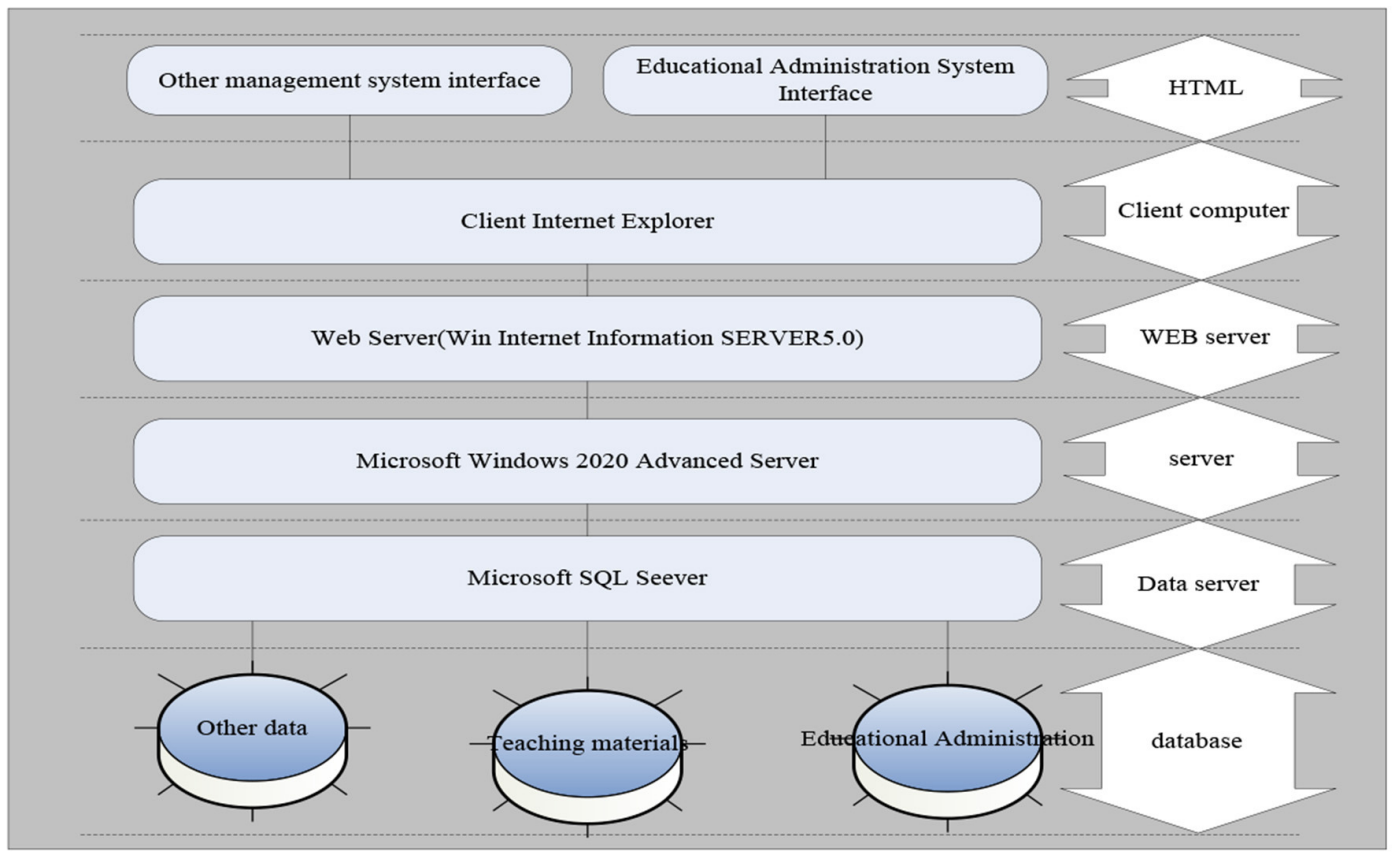
The overall structure of the logic of the system.

The system is a large-scale network and distributed database application system, located on the server. On the one hand, the graphical user interface is located at one end of each business operator's client, and the business configuration program is located on the application server. The overall logical structure of the system is shown in Figure 4.

### System Function Module

**Figure 5:**
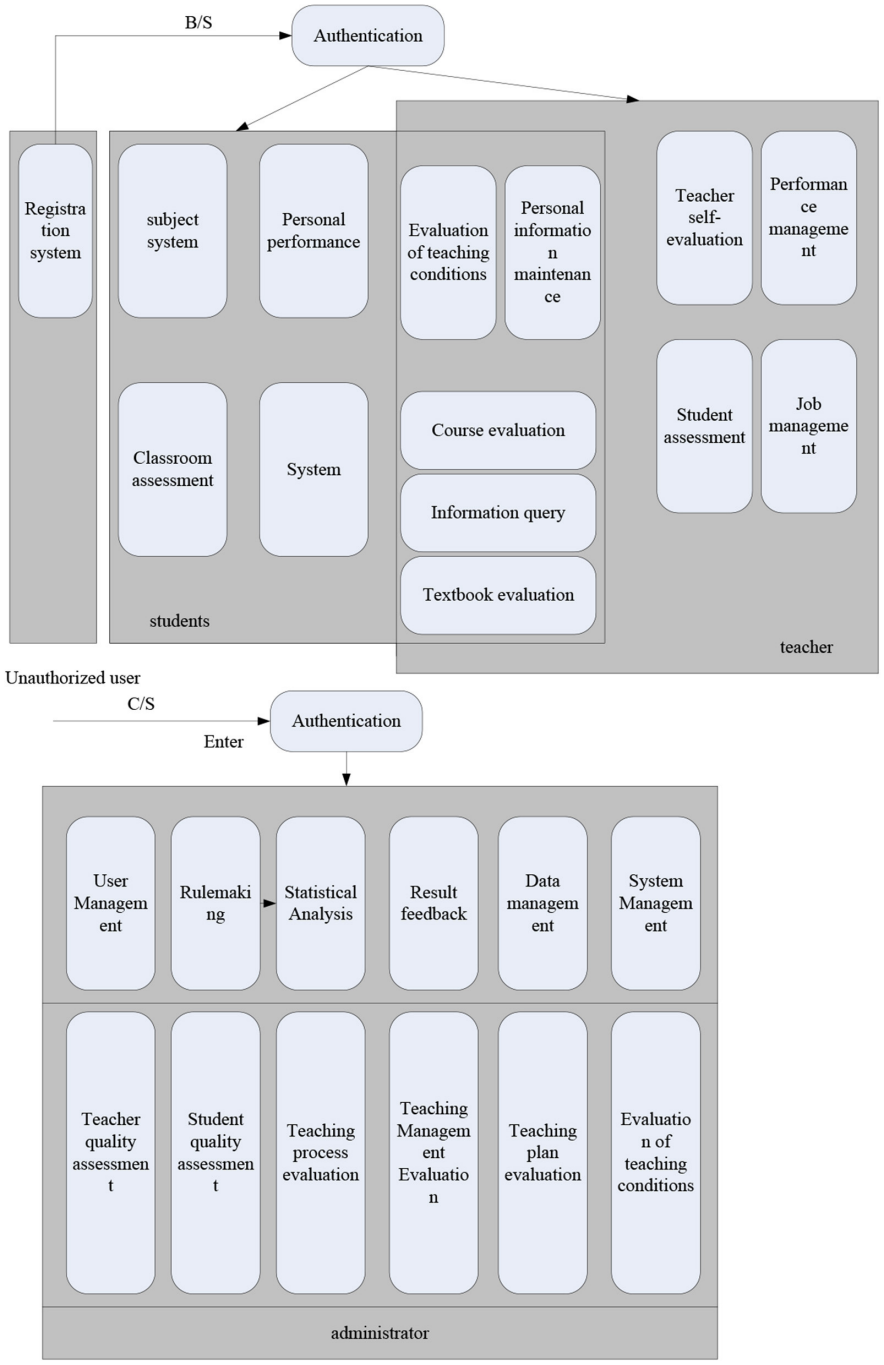
System function module diagram.

Figure 5 is a diagram of system function modules, showing the specific implementation principle of the system.

### Weight Model of Teaching Quality Evaluation

The judgment matrix is the basic information of the analytic hierarchy process, and it is also an important basis for calculating the weight of each element.

After establishing the hierarchical structure, the relationship of the elements between the upper and lower levels is determined. Supposing that for criterion H, there are *n* elements *A*_1_, *A*_2_,…, *A*_*n*_ in the next layer. A certain element *H* of the previous layer is used as the judgment criterion, and the *n* elements of the next layer are compared to determine the element value of the matrix. The form is as follows:


(1) $$\begin{array}{l} A=\left[\begin{array}{cccc} a_{11} & a_{12} & ... & a_{1n}\\ a_{21} & a_{22} & ... & a_{2n}\\ ... & ... & ... & ...\\ a_{n1} & a_{n2} & ... & a_{nm} \end{array}\right] \end{array}$$

In matrix *A*, a represents the relative importance of factor *A*_*i*_ to *A*_*j*_ from the perspective of judgment criterion *H*. If it is assumed that the weights of factors *A*_1_, *A*_2_…, *A*_*n*_ are, respectively, *w*_1_, *w*_2_,…, *w*_*n*_ under criterion *H*, that is:


(2) $$\begin{array}{l} w={\left(w_{1,}w_{2},...,w_{n}\right)}^{T} \end{array}$$

Then *a*_*ij*_ = *w*_*i*_/*w*_*j*_, obviously *a*_*ij*_ satisfies:


(3) $$\begin{array}{l} a_{ij}=1 \end{array}$$

(4) $$\begin{array}{l} a_{ij}=1/a_{ji} \end{array}$$

(5) $$\begin{array}{l} a_{ik}\cdot a_{kj}=a_{ij} \end{array}$$

Matrix *A* is called the judgment matrix.

The scale of the judgment matrix. The element *a* in the judgment matrix is a quantitative scale indicating the relative importance of two elements, called the judgment scale, and its values and meanings are shown in Table 3.

**Table 3:** The judgment standard table.

**Judgment scale**	**Definition**	**Judgment scale**	**Definition**
1	*a_*i*_* is as important as *a_*j*_* for H	7	*a_*i*_* and *a_*j*_* are much more important to *H*
3	For *Hai, a_*i*_*, and *a_*j*_* are slightly more important	9	For *Hai, a_*i*_*, and *a_*j*_* are absolutely important
5	*a_*i*_* and *a_*j*_* are important to *H*	2, 4, 6, 8	Between the above two adjacent judgment scales

Calculating the relative weight of each element.

When using the AHP method to evaluate, we need to know the relative importance of *A*_*i*_ with respect to *H*, that is, the weight of *A*_*i*_ with respect to *h*. Asking for $W={\left(w_{1},w_{2},...,w_{n}\right)}^{T}$.

Now it is known that *A* = (*a*_*ij*_)_*n*×*n*_ = [_*w*_*i*_/*w*_*j*_]*n*×*n*_,


(6) $$\begin{array}{l} \left[\begin{array}{cccc} w_{1}/w_{1} & w_{1}/w_{2} & ... & w_{1}/w_{n}\\ w_{2}/w_{1} & w_{2}/w_{2} & ... & w_{2}/w_{n}\\ ... & ... & ... & ...\\ w_{n}/w_{1} & w_{n}/w_{2} & ... & w_{n}/w_{n} \end{array}\right]\;\left[\begin{array}{c} w_{1}\\ w_{2}\\ ...\\ w_{n} \end{array}\right]=n\;\left[\begin{array}{c} w_{1}\\ w_{2}\\ ...\\ w_{n} \end{array}\right] \end{array}$$

(7) $$\begin{array}{l} AW=Nw \end{array}$$

It can be seen that *n* is an eigenvalue of matrix *A*, and *W* is the eigenvector of matrix A corresponding to eigenvalue *n*. From the characteristic equation of matrix *A*:


(8) $$\begin{array}{l} |A-\lambda E|=0 \end{array}$$

The following uses the square root method to calculate the maximum eigenvalue of the matrix and the corresponding eigenvector:

(1) Calculating the product *M*_*i*_ of each row element of matrix *A*
  (9) $$\begin{array}{l} M_{i}=a_{i1}a_{i2}...a_{in}=\overset{n}{\underset{j=1}{\prod }}a_{ij}\;i=1,2,...,n \end{array}$$
(2) Calculating the *n*th root of *M*_*i*_ $w_{i}^{\left(0\right)}$
  (10) $$\begin{array}{l} w_{i}^{\left(0\right)}={\left(\overset{n}{\underset{j=1}{\prod }}a_{ij}\;\right)}^{\frac{1}{n}}\;i=1,2,...,n \end{array}$$
(3) Pair vector
  (11) $$\begin{array}{l} w_{i}^{\left(0\right)}={\left(w_{1}^{\left(0\right)},w_{2}^{\left(0\right)},...,w_{n}^{\left(0\right)}\right)}^{T} \end{array}$$

For normalization, that is to say


(12) $$\begin{array}{l} w_{i}=w_{i}^{\left(0\right)}/\overset{n}{\underset{i=1}{\sum }}w_{i}^{\left(0\right)}\;i=1,2,...,n \end{array}$$

Thus, another vector $w_{i}^{\left(0\right)}={\left(w_{1}^{\left(0\right)},w_{2}^{\left(0\right)},...,w_{n}^{\left(0\right)}\right)}^{T}$ is obtained.

Calculate the maximum eigenvalue λ_max_ of *A* by:


(13) $$\begin{array}{l} Aw=\lambda_{\max}w \end{array}$$

(14) $$\begin{array}{l} Aw=\left[\begin{array}{c} \overset{n}{\underset{j=1}{\sum }}a_{1j}w_{j}\\ \overset{n}{\underset{j=1}{\sum }}a_{2j}w_{j}\\ \overset{n}{\underset{j=1}{\sum }}a_{nj}w_{j} \end{array}\right]=\left[\begin{array}{c} \lambda_{\max}w_{1}\\ \lambda_{\max}w_{2}\\ ...\\ \lambda_{\max}w_{n} \end{array}\right] \end{array}$$

It can get:


(15) $$\begin{array}{l} \lambda_{\max}=\frac{1}{n}\overset{n}{\underset{i=1}{\sum }}\frac{{\left(Aw\right)}_{i}}{w_{i}} \end{array}$$

After getting λ_max_, it should check whether it is consistent, that is, the appraiser's appointment. In the process of judgment and comparison, they maintain the consistency of the thinking process. However, due to diversification, unilateralism, multiple evaluation factors, and large-scale evaluators, not all judgments need to be completely consistent. Therefore, in order to ensure that a reasonable conclusion can be drawn from the use of the hierarchical system, it is necessary to try to fit the judgment model. We introduce a random consensus ratio.

Calculating the consistency index *CI* (Consistence Index)


(16) $$\begin{array}{l} CI=\frac{\lambda_{\max}-n}{n-1} \end{array}$$

There is a hierarchical model composed of target layer A, criterion layer C, and plan layer P. The relative weight of criterion layer C to target layer A is:


(17) $$\begin{array}{l} w_{i}^{\left(1\right)}={\left(w_{1}^{\left(1\right)},w_{2}^{\left(1\right)},...,w_{n}^{\left(0\right)}\right)}^{T} \end{array}$$

The weight vector of the sub-criteria layer S to the criteria of the criterion layer C as the criterion is (where the weight of the undominated element is zero):


(18) $$\begin{array}{l} w^{\left(2\right)}={\left(w_{1l}^{\left(2\right)},w_{2l}^{\left(2\right)},...,w_{nl}^{\left(2\right)}\right)}^{T}\;L=1,2,...,k \end{array}$$

Then the weight of the sub-criteria layer S to the target layer A is:


(19) $$\begin{array}{l} S_{ij}=w_{i}^{\left(1\right)}w_{jl}^{\left(2\right)} \end{array}$$

For the consistency check of the entire hierarchical model, it can be carried out as follows: Sub-criteria level S vs. target level A


(20) $$\begin{array}{l} CI_{s}=\left(CI_{s}^{1},CI_{s}^{2},...,CI_{s}^{n}\right)w^{\left(2\right)}\;n=1,2,...,k \end{array}$$

(21) $$\begin{array}{l} RI_{s}=\left(RI_{S}^{1},RI_{S}^{2},...,RI_{S}^{n}\right)w^{\left(2\right)}\;n=1,2,...,k \end{array}$$

(22) $$\begin{array}{l} CR_{s}=CI_{s}/RI_{s} \end{array}$$

## Methods of Interactive Teaching

### Psychological Foundation

Constructivist theory, as one of the most influential theories in educational practice, originated from the theory of children's thinking development. It began to appear in the late seventies. Like the philosophy of learning theory, its theoretical basis is not to accept passive but to accept active ideological knowledge, while active learning is considered to be based on knowledge. Constructivism is a branch of cognitive philosophy, which believes that individuals gradually establish an understanding of the world through the connection between experience and environment (including themselves). According to constructivism, four interrelated key factors—teacher, student, classroom, and environment will affect the learning process. Based on these classic constructivist theories, the basic point of constructivism is to deny the existence of objective knowledge, and to regard knowledge as students' self-explanation of reality, which students obtain from their own experience. That is to say, the only way to learn to acquire knowledge is to associate and identify new knowledge based on one's accumulated experience, and to establish the meaning of knowledge through interaction with the surroundings. Therefore, we need to give full play to the benign interaction between teachers and students in the classroom, teachers not only play the roles of knowledge managers and classroom managers in traditional classrooms, but also guide students to actively participate in classroom interaction as facilitators, negotiators, and facilitators, so that students no longer passively receive information. In the teaching process, we must give full play to the initiative and creativity of teachers and students. Teachers learn actively and students learn independently. In the teacher–student relationship, integration is a form of interdisciplinary communication, and the teacher–student is a key part of establishing cooperation. Constructivism also emphasizes the importance of real teaching scenes. Classroom teaching should be carried out in the context of teacher–student interaction and based on a good teacher–student relationship. This situation is closely related to students' lives and is of great significance. In this case, teachers and students will interact, communicate, and collaborate in the classroom to complete the knowledge building process. In general, active learning, the construction of knowledge and context, and the construction of interactive promotion have a great impact on the traditional teaching model, and also laid a solid foundation for the interactive classroom model and “deductive subjectivity.” Leading ideology, interaction, context, etc., can provide effective guidance for the interactive classroom model (Xindong and Jingguo, 2019).

### Student Learning Behavior Data

**Figure 6:**
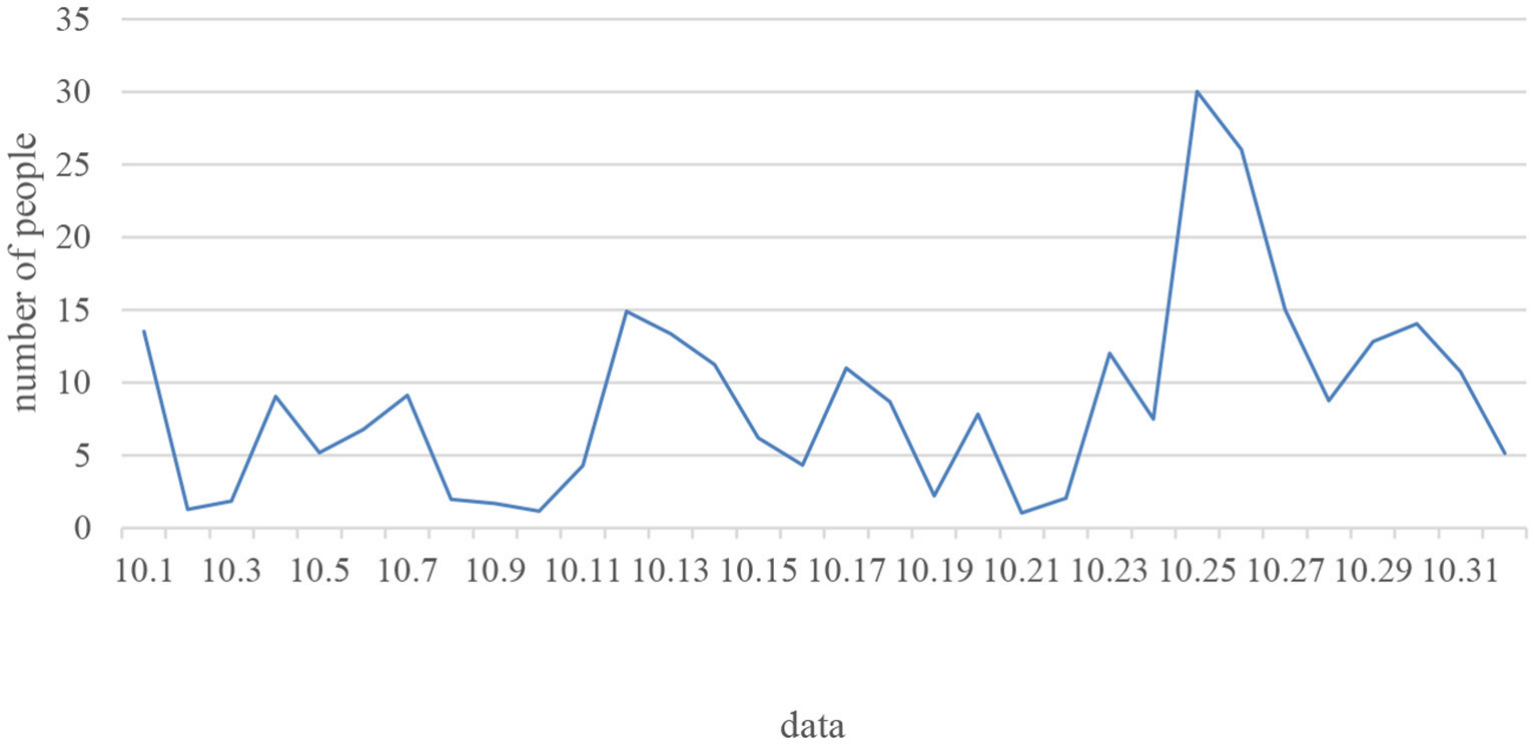
Number of visits to the student course page.

In order to conduct a more detailed analysis of the teaching effect of the teaching mode and the participation of students. At the beginning of October, 11 students were added to the class for ordinary online course learning, and the interactive teaching mode is scheduled to be implemented on the 23rd. After the end of the practice, the author retrieved the students' learning data from the background, and conducted an in-depth analysis of the application effects. First, the overall number of course visits of the students in October was analyzed (Figure 6). Figure 6 shows that the number of student visits is not the same (Hongsermeier et al., 2017). However, in the three consecutive days after October 23, the number of visits gradually increased, and the number of visits on the 25th was the most. It can be concluded that after the interactive teaching mode of this course, the autonomy of students in learning has been greatly improved. In other online learning, students have some visits, but the overall visit rate is low. Secondly, the number of students' discussions is also counted. Through analysis of data, it is found that students have discussed during the learning process. However, there are not many discussions. It is found through the data that the students' discussions are very active. By checking the specific discussion time of the students, it is found that the students are discussing knowledge before, during, after, and outside class. Therefore, it can be found that through the live broadcast of the classroom, the enthusiasm of students for autonomous learning has been improved. Students can discuss more frequently, learn from each other, and broaden the dimension of knowledge between students, which is conducive to better learning for students. Finally, by checking some of the student's messages, it is found that students like the interactive teaching mode. Listening to the lectures of famous school teachers through live broadcast in class can further broaden students' professional knowledge and improve learning efficiency. Tables 4, 5 show the discussion of students' online courses and the discussion of students' live courses (Borchardt and Bozer, 2017).

**Table 4:** Discussions of students' online courses.

**Student name**	**Total number of discussions**	**Post a discussion**	**Reply to discussion**	**Fraction**
A	10	3	7	Qualified
B	12	8	4	Qualified
C	11	6	5	Qualified
D	1	1	0	Qualified
E	4	4	0	Qualified
F	0	0	0	Qualified
G	2	2	0	Qualified
H	8	6	2	Qualified
I	14	8	6	Qualified
J	13	11	2	Qualified
K	20	9	14	Qualified

**Table 5:** Student course discussion.

**Student name**	**Total number of discussions**	**Post a discussion**	**Reply to discussion**	**Fraction**
A	53	25	28	Qualified
B	60	15	45	Qualified
C	50	6	44	Qualified
D	43	20	23	Qualified
E	4	4	0	Qualified
F	37	15	22	Qualified
G	70	34	36	Qualified
H	55	24	31	Qualified
I	47	12	35	Qualified
J	13	11	2	Qualified
K	59	21	38	Qualified
L	52	2	50	Qualified
M	60	34	30	Qualified
N	3	3	0	Qualified
O	25	10	15	Qualified
P	63	11	52	Qualified
Q	17	10	7	Qualified

**Figure 7:**
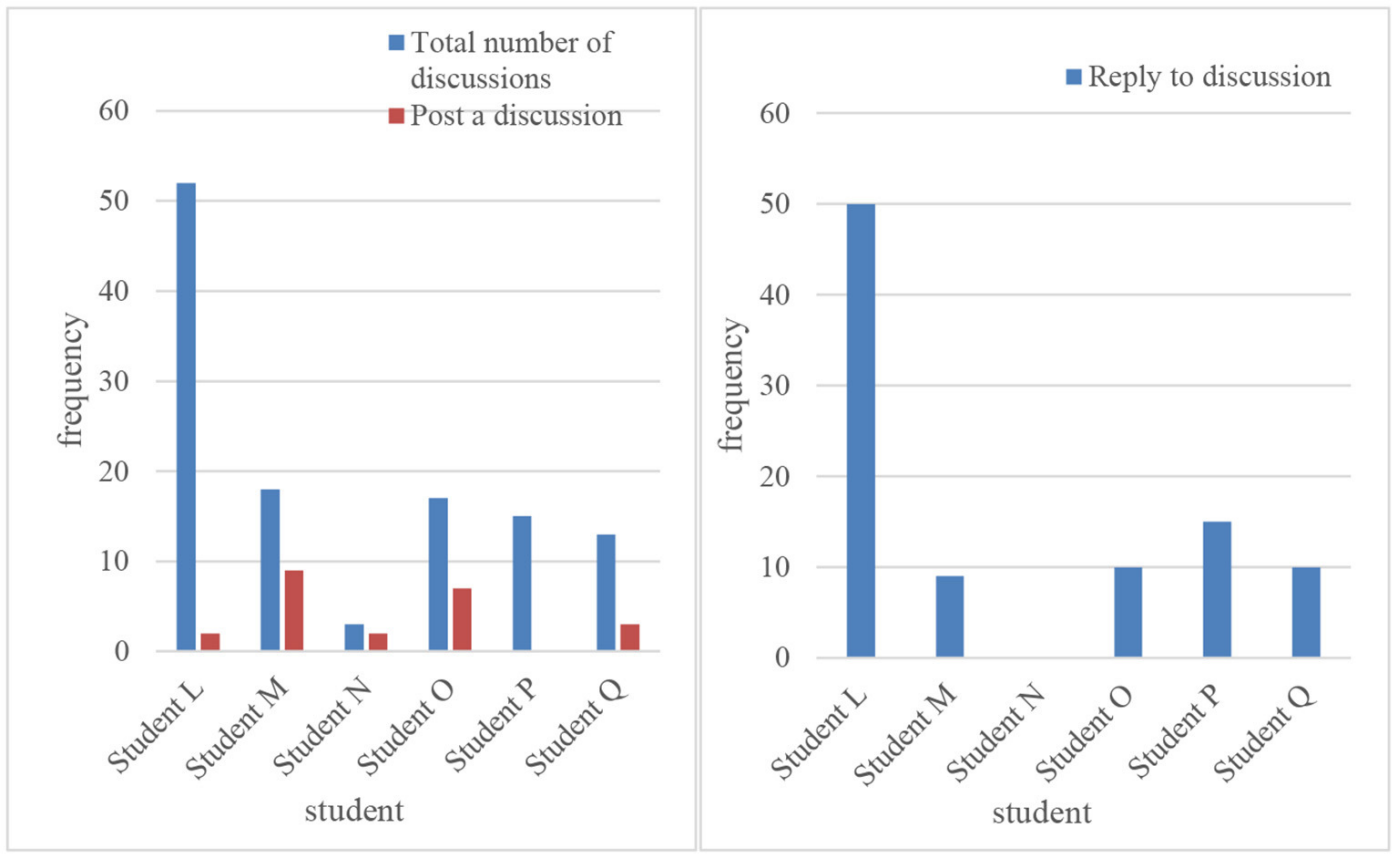
Discussion of students' online courses.

**Figure 8:**
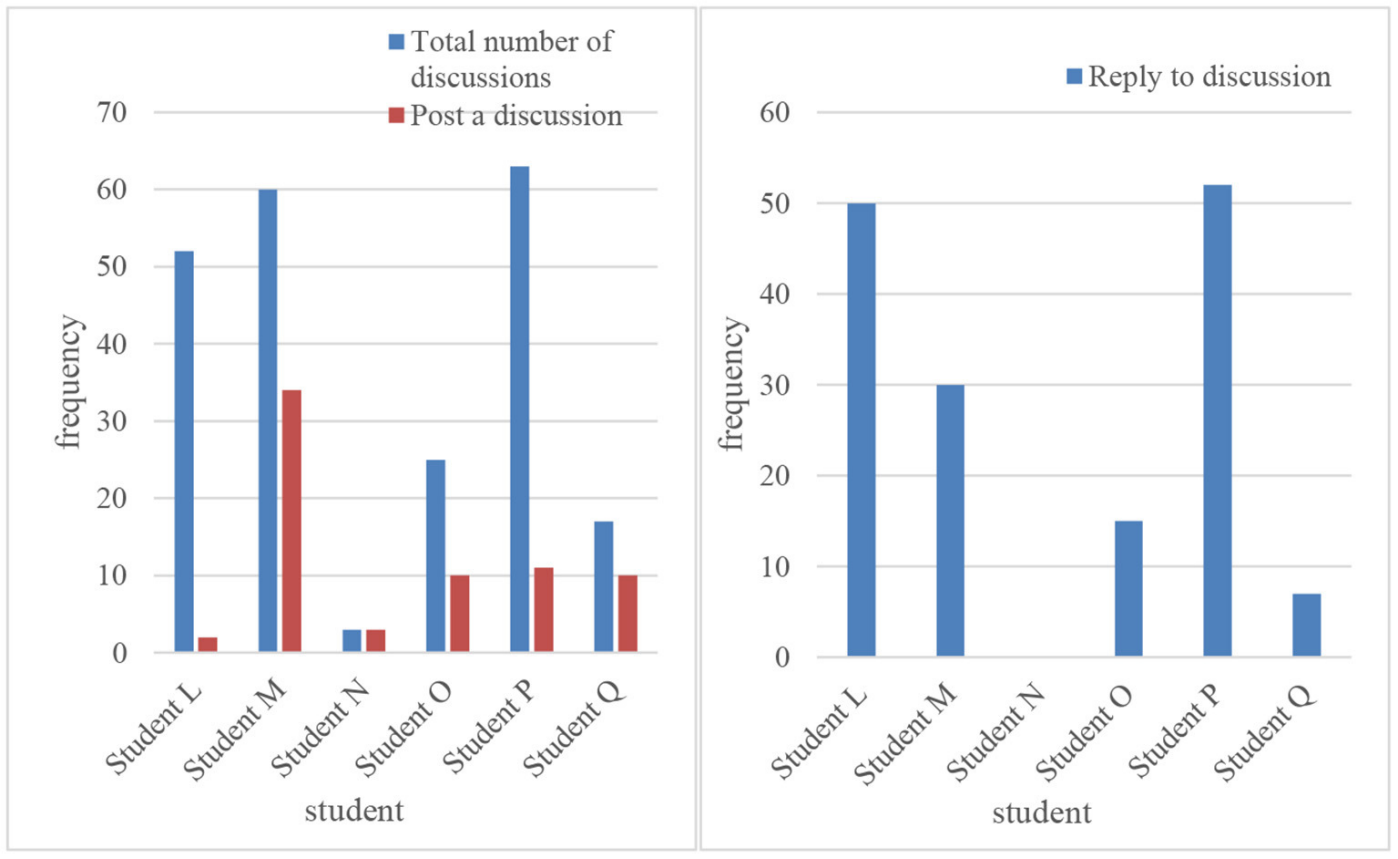
Comparison of the number of student indicators.

Through live interviews with teachers and students and analysis of background data, it is concluded that the vast majority of students agree with this model. This model can be used for pre-class preview, interactive participation in class, mutual learning, and exchange of tests after class. In classroom teaching, students' brains can be mobilized for active thinking. In terms of group collaboration, student participation is also very active. By studying the courses of teachers of other schools, students get different learning experiences. In the process of interacting with students of other schools, students boldly express their ideas and collide with each other in thought, and they all gain a sense of accomplishment and realize their self-worth (Podges et al., 2017). Through this model, teachers have realized the recycling of teaching resources, and teachers can have more energy and time to devote themselves to teaching. Figures 7, 8 are comparison diagrams of students' course discussions (Acharya et al., 2017).

### Problems of University Public Classroom Interaction

Taking English as an example, the traditional college public English classroom ignores the importance of language context, which makes English teaching deviate from the natural laws of language learning, and language knowledge and application ability are separated, students' low interest in classroom participation, low participation enthusiasm, and boring classroom atmosphere are one of the important reasons for the low effectiveness of students' language learning (Farashahi and Tajeddin, 2018). Teachers should improve the means of creating classroom interaction situations, so that students have an immersive experience in the classroom, touch students' emotions and attitudes in using foreign languages, stimulate students' interest and enthusiasm for participation, and make classroom interactive activities more smooth and efficient. The law of second language acquisition shows that the meaning of words and sentences in English learning are not isolated, they always exist depending on a certain situation. The meaning of language in human activities and situations, foreign language teaching should enable students to complete the meaning construction of the target language through real situations and meaningful interactions. Teachers should work hard to construct a real and meaningful language situation, similar to real social situations and life situations, place classroom teaching in real language practice, change the mode of only focusing on teaching in traditional teaching, and form a classroom based on practical exercises. This can encourage students to construct the meaning of knowledge in real language situations in classroom interaction (Ahmad et al., 2018).

Classroom atmosphere refers to various psychological and social atmospheres in the classroom, such as the teacher's degree of field control, flexibility, anxiety, control, initiative, and enthusiasm. Related scholars discussed the relationship between the mental state of the classroom and the influence of teaching, and believed that the mental atmosphere in the classroom is a group emotional state that can realize the smooth progress of the classroom teaching process (Wuryan, 2017). A healthy and positive psychological atmosphere in the classroom can be a good reason to improve teaching effectiveness. The psychological atmosphere of a negative or even confrontational classroom will make teachers and students find or think that the classroom is a burden.

**Figure 9:**
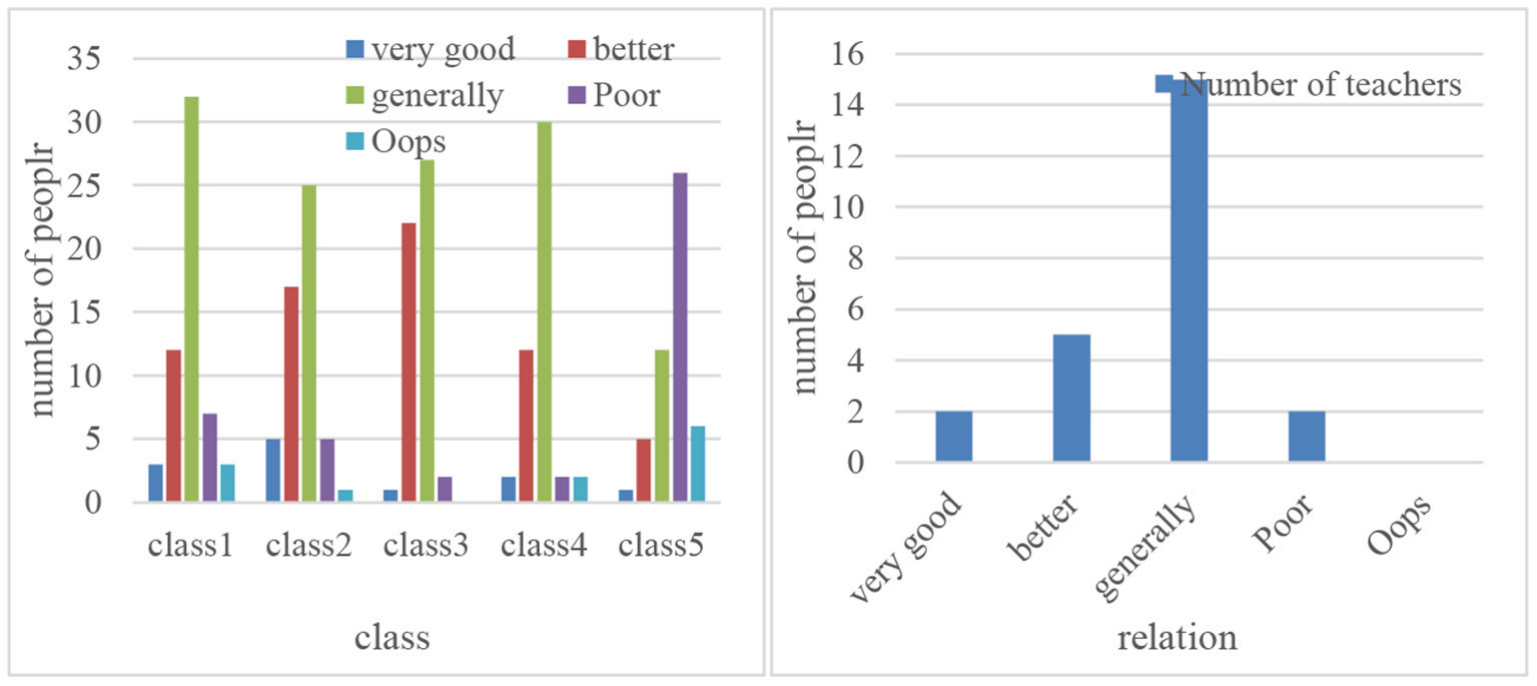
Comparison of teacher–student relationship.

The main way for college students to acquire knowledge is to allow teachers and students to focus on textbooks and effectively transmit them throughout the classroom. In this way, classroom teaching has become the most basic form of university education. The classroom teaching concept advocated by traditional teaching methods is that teachers simply share information and students receive information mechanically. Modern education attaches great importance to the building of students' ability and the establishment of new knowledge. How to create conditions, improve teaching methods, implement effective and harmonious teacher–student interaction, and transform traditional passive receptive learning into active participation and exploratory learning are major issues that modern teaching needs to solve. The teaching process is a process of communication. The interpersonal communication between teachers and students runs through the entire teaching process. Harmonious teacher–student relationship plays an important role in classroom interaction, and it is also the basic information needed to realize teaching and learning and teacher–student communication (Omar, 2017). Observed on the spot in college public English classrooms, most of the public English classroom teaching has serious dominance, and there is a lack of meaningful interaction between teachers and students. The teacher presets the teaching route in advance, and the teacher controls the course of the class with the fixed questions set in advance. Students dare and do not want to participate in classroom discussions and interactions, and passively participate in classroom teaching. Teachers appear in the classroom as a controller and knowledge authority. There is a serious lack of sincere emotional interaction between teachers and students, and the relationship between teachers and students is antagonistic and unequal. Figure 9 is a comparison diagram of the relationship between teachers and students (Filho and Paula, 2017).

**Figure 10:**
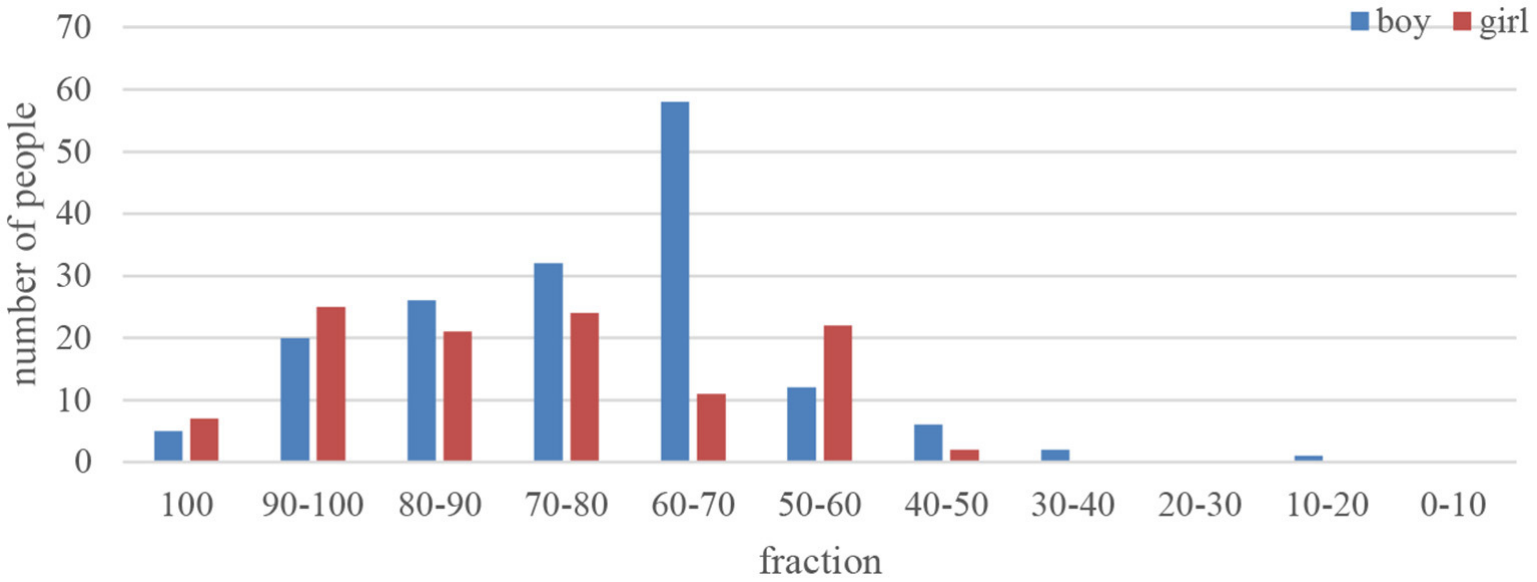
Self-evaluation diagram of learning subjectivity.

The student lacks subjectivity, and the student subject emphasizes the status of students in the process of teaching and understanding. Student subjectivity means that students are active knowledge masters and explorers in the teaching process; it requires student development as the center, respect, trust, and based on the initiative of students, and discover problems actively and independently, and under the leadership of the teacher, think of ways to solve the problem. Controversy about whether the teaching in the classroom is the main body of the teacher or the main body of the student has actually been heard in academia. Those who hold the teacher subject theory believe that the teacher is the only subject in the teaching process, emphasizes the teacher's knowledge authority status, and emphasizes the use of classroom teaching to instill knowledge into the students. The students, as passive recipients of education, are in a position of obedience and acceptance (Centrella-Nigro and Alexander, 2017). China has respected this view of teachers since ancient times, and has exaggerated the status of teachers too much. This view of education that only pays attention to indoctrination regards students as the container of knowledge, and regards teachers as the absolute authority of knowledge, ignoring the subjective initiative of students, thus inhibiting students' free development and active construction of knowledge meaning. Most of the current college public English teachers hold a teacher-centered education concept. In such a traditional teaching model, teachers regard themselves as the controller of the classroom and dominate the process and methods of the classroom. The teacher decides what students should do in the classroom, the time of interaction and the form of interaction, and the teacher can control the students' classroom reflection. Although teachers' leadership and control in the classroom are essential, under such a model it is easy to cause students to over-rely on teachers (Kim et al., 2017). Students cannot experience practical learning but habitually pay attention to teachers. Especially in large-class classes such as university public English classes, the opportunity to speak becomes less and most students choose to remain silent and very few students are willing to take the initiative to speak. In this traditional role of teachers and students, the classroom atmosphere is lifeless, the classroom forms and activities are monotonous, students have less power to speak, and the actual participation is low. As shown in the previous teaching situation, the teacher appears in the classroom as a knowledge authority, focusing on completing the established teaching route and plan, ignoring the student's foundation and understanding, not paying attention to creating a real teaching context, and focusing on the meaning of grammar and words, etc., language knowledge, and often use punishment and threats to manage students. This is an important manifestation of the lack of subjectivity of students. It is also one of the key factors that cause the low level of classroom interaction and the dull interaction atmosphere. Figure 10 shows the self-assessment of students' subjectivity.

## Discussion

The efficiency of classroom interaction depends on the level of teacher's classroom control ability. Classroom control is the core of education control. It is the process by which teachers consciously guide, restrain, and adjust student behavior (including their own behavior) in order to achieve the goal of classroom teaching. Classroom interactive control strategy refers to the process of consciously guiding, restraining, and adjusting the behavior of teachers and students when they use interactive teaching methods in the classroom. In the actual college English teaching, the teacher's control strategy is relatively scarce, resulting in the low efficiency of actual classroom interaction.

Most university public teachers control the classroom through language, and the teacher's words determine the quality of the entire classroom. Good teachers and teaching feedback can not only provide students with effective and easy-to-understand language input, but also encourage students to make more language output. Teachers' questioning is an important part of the teacher's discourse, and the waiting time of students after the teacher's questioning plays an important role in the quality of students' language output. If students are assigned to answer questions, the teacher's average waiting time is no <2 s. If there is no response after 2 s, the teacher will answer separately or ask other students to answer (Loveland and Love, 2017). In some classrooms, if the teacher tries to extend the waiting time by 3–5 s, more students will participate in class activities, and the average response time of students will increase by 40. Teacher feedback and error correction are also important components. There are two types of teacher feedback: positive feedback and negative feedback. A large number of studies have found that positive feedback is better than negative feedback in improving students' motivation for learning and participation. Studies have also shown that students expect effective feedback from teachers. In the classroom, teachers often use feedback strategies such as meaning negotiation and discourse repair in order to keep the teacher–student communication smooth and to promote the further language output of the teache–student communication topics. Meaning negotiation includes comprehension verification, confirmation, and clarification methods, while discourse restoration includes repetition, restatement, and other strategies. Meaning negotiation and discourse restoration strategies have completely different roles in the classroom (Schafer, 2019). In daily conversations, people often use confirmation and clarification feedback methods to solve problems and misunderstandings in communication, but in traditional college public English classrooms, the most commonly used by teachers is the feedback strategy of understanding and verification. The advantage of this feedback strategy is to ensure the teacher's absolute control in classroom interaction (Kris, 2017). After explaining the knowledge content, the teacher simply uses the means of confirmation and verification to give feedback, because in the university public English classroom, the traditional grammar-centered teaching method is still dominant. Teachers often ignore the use of feedback methods such as confirmation and clarification, especially in the process of improving students' language ability (Kitayama, 2017). Because the feedback means of understanding and verification can easily lead to unidirectional communication in the classroom, only the information input from the teacher to the student does not achieve the purpose of the student's meaningful language output (Balu and Mukherjee, 2021). Teachers need to be aware of the shortcomings of purely understanding and verification feedback methods, and consciously increase feedback methods such as confirmation and verification, to change the traditional one-way grammar-centered classroom interaction environment, and to provide students with sufficient language input while also having enough language opportunities to output opportunities, thereby improving their language output and communication skills (Verner et al., 2021).

## Conclusion

All classroom teaching activities are actually a kind of psychological communication activities between multiple disciplines. In these types of communication activities, frequent and close relationships occur between teachers and students, between students and students, and between teachers and teachers, forming a complex relationship system. The relationship between the various components of the system is not simple. The “additional” relationship is an organic “integration” relationship. Therefore, teachers are no longer just providers of professional knowledge, and students are no longer passive receivers. On the contrary, they emphasize the acquisition of knowledge through various forms of interaction, and they emphasize to provide students with multiple opportunities. In interactive classroom teaching, factors such as teachers, students, classrooms, and environment are no longer the only ones, but they influence each other, relate to each other, and promote each other. Therefore, in this interactive classroom teaching, students are always the core elements of the classroom, and teachers are the guides of classroom activities. Teachers and students interact in many ways, which embodies the teaching concept of “Teacher as the main body and students as the main body.” Teaching quality monitoring is currently a hot topic in the development of education in our country, and many schools have carried out evaluation and self-evaluation. However, because the teaching quality monitoring is still in the review stage, the teaching quality monitoring model has not been fully and rapidly expanded. The innovation of the article lies in the introduction of advanced object-oriented visual modeling technology, and the development of a teaching quality monitoring system, based on in-depth research on object-oriented modeling ideas, semantics, and characteristics. And in the process of its development, a teaching quality-oriented teaching system was established, and the characteristics and current status of the new teaching quality monitoring system were introduced. The model focuses on the key concepts and factors of monitoring teaching quality, and uses cases to drive the entire development process. It uses standard graphic elements to describe the static structure and dynamic nature of the system, and realizes iterative and incremental development of the system. Although we have established a relatively complete teaching quality monitoring system, with the optimization of the teaching quality evaluation index system, there are still some problems that look forward to the further development of network application technology and further in-depth research for continuous improvement. It is an excellent visual modeling language in object-oriented analysis and design, but it has not proposed a better visualization scheme in the realization of object-oriented program code. Based on Rose, adding visualization program code implementation modules to provide an extended modeling environment will be a research direction.

## Data Availability Statement

The original contributions presented in the study are included in the article/supplementary material, further inquiries can be directed to the corresponding author/s.

## Author Contributions

KC: writing—original draft preparation.

## Conflict of Interest

The author declares that the research was conducted in the absence of any commercial or financial relationships that could be construed as a potential conflict of interest.

## Publisher's Note

All claims expressed in this article are solely those of the authors and do not necessarily represent those of their affiliated organizations, or those of the publisher, the editors and the reviewers. Any product that may be evaluated in this article, or claim that may be made by its manufacturer, is not guaranteed or endorsed by the publisher.
